# Pharmacological Targets of Kaempferol Within Inflammatory Pathways—A Hint Towards the Central Role of Tryptophan Metabolism

**DOI:** 10.3390/antiox9020180

**Published:** 2020-02-21

**Authors:** Stefanie Hofer, Simon Geisler, Rebecca Lisandrelli, Hieu Nguyen Ngoc, Markus Ganzera, Harald Schennach, Dietmar Fuchs, Julian E. Fuchs, Johanna M. Gostner, Katharina Kurz

**Affiliations:** 1 Institute of Medical Biochemistry, Biocenter, Medical University of Innsbruck, Innrain 80, 6020 Innsbruck, Austria; stefanie.hofer@uibk.ac.at (S.H.); rebeccalisandrelli@gmail.com (R.L.); johanna.gostner@i-med.ac.at (J.M.G.); 2 Institute of Pharmacy/Pharmacognosy, University of Innsbruck, Innrain 80 - 82/IV, 6020 Innsbruck, Austria; hieu.nguyen-ngoc@student.uibk.ac.at (H.N.N.); markus.ganzera@uibk.ac.at (M.G.); 3 Institute of Biological Chemistry, Biocenter, Medical University of Innsbruck, Innrain 80, 6020 Innsbruck, Austria; simon.geisler@i-med.ac.at (S.G.); dietmar.fuchs@i-med.ac.at (D.F.); 4 Central Institute of Blood Transfusion and Immunology, University Hospital, Anichstrasse 35, 6020 Innsbruck, Austria; harald.schennach@tirol-kliniken.at; 5 Department of Medicinal Chemistry, Boehringer Ingelheim RCV GmbH & Co KG, Dr. Boehringer-Gasse 5- 11, 1120 Vienna, Austria; julian.fuchs@boehringer-ingelheim.com; 6 Department of Internal Medicine II, Infectious Diseases, Pneumology, Rheumatology, Medical University of Innsbruck, Anichstrasse 35, 6020 Innsbruck, Austria

**Keywords:** kaempferol, indoleamine 2,3-dioxygenase, tryptophan, neopterin, antioxidant, phytochemical

## Abstract

The flavonoid kaempferol is almost ubiquitously contained in edible and medicinal plants and exerts a broad range of interesting pharmacological activities. Interactions with central inflammatory processes can be exploited to treat or attenuate symptoms of disorders associated with chronic immune activation during infections, malignancies, and neurodegenerative or cardiovascular disorders. Many drugs, phytochemicals, and nutritional components target the catabolism of the essential amino acid tryptophan by indoleamine 2,3-dioxygenase 1 (IDO-1) for immunomodulation. We studied the effects of kaempferol by in vitro models with human peripheral blood mononuclear cells (PBMC) and THP-1 derived human myelomonocytic cell lines. Kaempferol suppressed interferon-γ dependent immunometabolic pathways: Formation of the oxidative stress biomarker neopterin and catabolism of tryptophan were inhibited dose-dependently in stimulated cells. In-silico docking studies revealed a potential interaction of kaempferol with the catalytic domain of IDO-1. Kaempferol stimulated nuclear factor kappa B (NF-κB) signaling in lipopolysaccharide (LPS)-treated THP-1 cells, thereby increasing the mRNA expression of interleukin (IL) 1 beta, tumor necrosis factor, and nuclear factor kappa B subunit 1, while IL6 was downregulated. Data suggest that concerted effects of kaempferol on multiple immunologically relevant targets are responsible for its immunomodulatory activity. However, the immunosuppressive effects may be more relevant in a T-cell dominated context.

## 1. Introduction

Kaempferol (3,4′,5,7-tetrahydroxyflavone) is a secondary plant metabolite with a hydroxy phenylchromenone structure belonging to the family of flavonoids. Like other flavonols, kaempferol is a product of the phenylpropanoid pathway [1]. Flavonoids are distributed nearly ubiquitously in the plant kingdom, as these compounds exert essential roles in the development, growth, and survival of plants. They are of major relevance in oxidative stress defense processes [2,3]. 

Kaempferol can be found in many edible and medicinal plants. In a study conducted between 1999 and 2005, a mean intake of 5 ± 1 mg kaempferol per day has been estimated using data derived from the European Food Safety Authority (EFSA) and the FLAVIOLA Food Composition Database, which contain information on dietary intake from 30,000 individuals aged 18–64 years from 14 European countries [4]. With a plant-rich diet, even higher intake values of up to 11 mg/day can be reached, as estimated in the prospective U.S. Nurses’ Health Study involving more than 60,000 women [5]. The fact that increased kaempferol intake is associated with a reduced risk to develop certain diseases has been investigated in numerous epidemiological studies [6]. This flavonoid is known to exert a broad range of pharmacologically relevant activities in humans, including the mediation of antioxidant, anti-inflammatory, antimicrobial, cardio-, and neuroprotective effects [6]. 

The ability of kaempferol to attenuate oxidative stress has been reported both in vitro and in vivo, involving direct and indirect mechanisms. Kaempferol scavenges different types of radicals, it inhibits reactive oxygen species (ROS)-generating enzymes and increases the expression of antioxidant enzymes [6,7,8]. 

Oxidative stress is a crucial trigger of inflammation and redox reactions participate in the perpetuation and termination of inflammation, as well as in the initiation of regenerative processes [9]. A fine-tuned regulation of the involved biochemical pathways is necessary to ensure an effective immune response, however, if control mechanisms fail, pathological conditions may develop. During immune response, activated T cells release the central cytokine interferon gamma (IFN-γ), which activates ROS production by NADPH oxidases [10,11] and triggers phagocytosis and antigen-presentation [12]. Its downstream signaling is further involved in the orchestration of several cross-regulated immunological pathways, including tryptophan (Trp) breakdown via indoleamine 2,3-dioxygenase (IDO-1), formation of neopterin via GTP-cyclohydrolase I (GTP-CH-I), and activation of nuclear factor κ-light-chain-enhancer of activated B cells (NF-κB)-dependent signaling cascades [9,13].

The catabolism of the essential amino acid Trp along the kynurenine (Kyn) axis was initially identified as a defense mechanism to restrict the growth of invading pathogens by nutrient deprivation [14]. Later on, the consequences of this mechanism on immunoregulation, which are caused by a suppression of T cell proliferation and differentiation, have been explored in more detail [15,16,17]. In many chronic diseases, like infections, autoimmune, or malignant disease, as well as in neurodegenerative and cardiovascular disorders, an accelerated Trp breakdown has been reported and goes along with elevated Kyn to Trp ratios (Kyn/Trp) in the blood of patients [18]. Moreover, dysregulation of the Trp catabolic route along the Kyn axis may be implicated in the pathophysiology of depression. [18,19]. In parallel, patients suffering from these chronic diseases often present with elevated concentrations of the oxidative stress marker neopterin. Neopterin formation coincides with Trp breakdown during cellular (Th1-type) immune activation, as both pathways are induced by IFN-γ [20]. Meanwhile, Kyn/Trp and neopterin are well-established biomarkers for disease monitoring [18,20]. Moreover, these immuno-biochemical pathways have emerged as interesting targets in the search of novel active pharmaceutical ingredients with immunomodulatory properties [21,22,23]. 

Another key pathway in inflammation is nuclear factor kappa-light-chain-enhancer of activated B cells (NF-κB) signaling, which is strongly induced by oxidative stress and thus is of outmost interest in the search for anti-inflammatory compounds [24]. NF-κB-signaling is tightly controlled by positive and negative regulatory mechanisms and depends on several interfering biochemical pathways [25]. The activation of NF-κB signaling leads to a variety of responses that are highly complex and cell type-specific, including the expression of several chemokines and cytokines [26] that trigger inflammation, the activation of pro-survival signaling cascades, as well as the upregulation of antioxidant enzymes like Mn-superoxide dismutase, or inducible nitric oxide synthase (iNOS) [27,28,29]. 

In this study, we investigated the effects of kaempferol on IFN-γ-mediated biochemical pathways and NF-kB signaling in human peripheral blood mononuclear cells (PBMC) and reporter cells derived from the myelomonocytic THP-1 cell line, which can be activated to a macrophage-like phenotype. Furthermore, potential interactions of kaempferol with IDO-1 were analyzed in an in-silico docking approach. Briefly, kaempferol interferes with both the immune-metabolic pathways (Trp breakdown and neopterin formation) as well as NF-κB signaling. Obtained results hint towards the importance of the Trp degrading enzyme IDO-1 as an intervention target for immunoregulatory phytochemicals.

## 2. Materials and Methods 

### 2.1. Chemicals

Kaempferol (Abcam, Cambridge, UK) was dissolved in 99.9% (*v/v*) ethanol. Quercetin (Sigma Aldrich, Vienna, Austria) and kaempferol 3-*O*-β-D-glucopyranosyl (1→2)-α-L-rhamnopyranoside, isolated from *Urceola rosea* leaves at the Institute of Pharmacy/Pharmacognosy University of Innsbruck, Austria [30], were dissolved in cell culture grade dimethyl sulfoxide. Phytohemagglutinin (PHA) and lipopolysaccharide (LPS) from *E. coli* O55:B5 were dissolved in phosphate buffered saline (PBS, all Sigma Aldrich, Vienna, Austria). Stocks of all chemicals were stored at –20 °C. 

### 2.2. Cell Culture

#### 2.2.1. Peripheral Blood Mononuclear Cells (PBMC) Isolation 

Peripheral blood mononuclear cells (PBMC) were isolated from the whole blood of healthy donors at the Central Institute of Blood Transfusion and Immunology, University Hospital of Innsbruck, Austria. Donors gave written consent that their blood might be used for scientific purposes in cases when it was not selected for transfusion. PBMC were separated by density centrifugation (Pancoll human, PAN Biotech, Aidenbach, Germany) [31]. Following the isolation process, cells were washed three times with PBS containing 1 mM ethylenediaminetetraacetic acid. 

Cells were maintained in Roswell Park Memorial Institute’s Medium (RPMI 1640) supplemented with 10% heat-inactivated fetal bovine serum (LifeTech, Vienna, Austria), 2 mM glutamine (Sigma Aldrich, Vienna, Austria), and 50 µg/mL gentamicin (Sigma Aldrich, Vienna, Austria) in a humidified atmosphere containing 5% CO_2_ for 48 h. For each of the experiments, PBMC were prepared freshly from different donors.

#### 2.2.2. Culture of Cell Lines 

The spontaneously immortalized human keratinocyte cell line HaCaT [32] (Cell Lines Service, Eppelheim, Germany) and the THP1-Blue and THP1-Blue-CD14 (Invivogen, San Diego, CA, USA) cell lines were maintained in RPMI supplemented with 10% fetal bovine serum (FBS) (*v/v*). In addition, THP1-Blue and THP1-Blue-CD14 cells were maintained in a medium containing 200 µg/mL of the selection antibiotic zeocin (Invivogen, San Diego, CA, USA). No antibiotic was added during the experiments. 

### 2.3. Cellular Antioxidative Activity (CAA) Assay 

1 × 10^4^ HaCaT cells in 100 µL/well were seeded in a 96-well microplate and incubated for 24 h. The measurements of relative changes in intracellular ROS were performed based on the original protocol of Wolfe and Liu [33], with several modifications [34], using the redox sensitive probe 2′,7′-dichlorofluorescin diacetate (DCFH-DA, Sigma Aldrich, Vienna, Austria) as substrate. In brief, cells were washed with pre-warmed PBS, and treated with 50 μL per well of 25 µM DCFH-DA in Hanks’ balanced salt solution (HBSS) for 1 h. DCFH-DA passes through the cell membranes and is subsequently deacetylated by intracellular esterases to DCFH, which cannot cross the cell membrane. Then, cells were treated with 50 µL kaempferol or kaempferol glycoside diluted in HBSS, resulting in final concentrations of 0.4 µM to 100 µM. Control cells were treated with buffer only. Additionally, the effect of the respective vehicle was tested. Maximum vehicle concentrations were 0.11% (*v/v*) DMSO for kaempferol glycoside and 0.10% (*v/v*) ethanol for kaempferol (EtOH, Carl Roth, Vienna, Austria) treated cells. 10 µM of quercetin was used as positive control. After an incubation period of 1 h, cells were washed twice with pre-warmed PBS and treated with 600 µM of the peroxyl radical generator 2-[(1-amino-1-imino-2-methylpropan-2-yl)diazenyl]-2-methylpropanimidamide (AAPH, Sigma Aldrich, Vienna, Austria) in HBSS. DCFH can be oxidized to its fluorescent derivative 2′7′- dichlorofluorescein (DCF) upon radical formation. After 45 min of incubation at 37 °C, the fluorescence signal of DCF (excitation 485 nm/emission 538 nm) was measured with a Tecan infinite F200 PRO plate reader (Tecan Group Ltd., Männedorf, Switzerland). DCF fluorescence is proportional to the level of intracellular ROS.

### 2.4. Cell Viability

Cells’ viability was estimated in parallel for all cell models using the same seeding and treatment concentrations and buffer/vehicle controls diluted in growth medium as mentioned in the individual experiments. The cells’ ability to metabolize the redox-dye resazurin to resorufin was applied as an indicator of cell viability. 10% (v/v) of CellTiter-Blue reagent (Promega, Mannheim, Germany) was added to the medium and cells were incubated at 37 °C. Incubation times were 50 min for HaCaT cells, 4 h for PBMC and 1h 15 min for THP1-Blue and THP1-Blue-CD14. The formation of resorufin was determined at 560 nm excitation/590 nm emission (Tecan infinite F200 PRO plate, Männedorf, Switzerland). Cell-free experiments showed that there was no unspecific interaction between the test compounds and resazurin itself (data not shown).

### 2.5. Treatment of PBMC, THP1-Blue, and THP1-Blue-CD14 Cells with Kaempferol

Isolated PBMC were seeded at a density of 1 × 10^6^ cells/mL, THP1-Blue and THP1-Blue-CD14 suspensions were seeded at a density of 0.5 × 10^6^ cells/mL in each well of a 6-well plate. Cells were stimulated or not with 10 µg/mL of the mitogen PHA (PBMC), 1 µg/mL LPS (THP1-Blue) or 100 ng/mL LPS (THP1-Blue-CD14) 30 min after treatment with the different concentrations of kaempferol. Cells were incubated for 24 and 48 h before harvesting cell supernatant by centrifugation (10 min, 1000 rpm) or estimation of other parameters (e.g., NF-κB/AP-1 activation in THP1-blue-CD14 cells). Data shown are from 24 h treatments only. Supernatants were transferred into fresh tubes and frozen at –20 °C until analysis. Cell pellets were used for RNA preparations or were washed twice with ice-cold PBS before preparation of protein lysates. 

### 2.6. Measurements of Tryptophan (Trp), Kynurenine (Kyn), and Neopterin

High-pressure liquid chromatography (HPLC) analysis was performed to determine Trp and Kyn concentrations according to Widner et al., with minor modifications [35]. 3-nitro-L-tyrosine (Sigma Aldrich, Vienna, Austria) was used as an internal standard. The Kyn/Trp ratio is expressed as µmol Kyn per mmol Trp and can be applied as an estimate of IDO-1 activity, if other immune activation markers are present in parallel [36]. Neopterin concentrations were measured using a commercially available enzyme-linked immunosorbent assay (ELISA) (Thermo-Scientific BRAHMS, Hennigsdorf, Germany) according to the manufacturers’ instructions. The limit of the detection of the assay is 2 nmol/L neopterin.

### 2.7. Determination of NF-κB/AP-1 Activation

THP1-Blue and THP1-Blue-CD14 cells are derived from the myelomonocytic THP-1 cell line and contain a nuclear factor kappa B (NF-κB)/activator protein 1 (AP1) inducible reporter construct for secreted embryonic alkaline phosphatase (SEAP). The latter also overexpresses CD14 for enhanced sensitivity toward toll-like receptor (TLR) stimulation.

Upon stimulation with LPS, SEAP expression was initiated. SEAP protein is secreted into the supernatant, and its activity can be measured colorimetrically at 635 nm by adding 10 µL cell supernatant to 100 µL QUANTI-Blue substrate (InvivoGen, San Diego, CA, USA). After an incubation period of 70 min at 37 °C protected from light, SEAP activity was determined at 625 nm using a Tecan infinite F200 PRO plate reader (Männedorf, Switzerland).

### 2.8. Western Blotting 

Cell pellets were washed twice with ice-cold PBS, and lysed in 150 µL lysis buffer (20 mM Tris-HCl (AppliChem, Darmstadt, Germany) pH 6, 150 mM NaCl (Carl Roth, Karlsruhe, Germany), 5 mM MgCl_2_ (Merck, Darmstadt, Germany), 5% (*v/v*) glycerol (Carl Roth, Karlsruhe, Germany), 0.5% Triton X-100 (Sigma Aldrich, Vienna, Austria)) and supplemented with protease inhibitors (10 µL/mL 100 mM phenylmethylsulfonyl fluorid (AppliChem, Darmstadt, Germany), 10 µL/mL 100 mM dithiothreitol (BioMol, Hamburg, Germany), 5 µL/mL 500 mM NaF (Fluka, Seelze, Germany), 0.5 µL/mL 10 mg/mL aprotinin, and 0.5 µL/mL 10 mg/mL leupeptin (both from Sigma, Vienna, Austria). After an incubation period of 30 min on a rotary incubator at 4 °C, lysates were centrifuged (20 min, 4 °C, 13,000 rpm) and supernatants were transferred into fresh tubes. Protein concentrations were estimated using Bradford’s reagent (BioRad, Hercules, CA, USA) according to manufacturer’s instructions. Equal amounts of protein (10–25 µg) heated in Laemmli sample buffer were loaded per lane, resolved by an 8% sodium dodecyl sulfate-polyacrylaminde gel electrophoresis (chemicals from Carl Roth, Karlsruhe, Germany) and transferred onto a polyvinylidene fluoride (PVDF) membrane (Immobilon-P, 0.45 μm pore size, Merck, Darmstadt, Germany) for Western blotting at 36 V for 1 h at 4 °C. After fixation with methanol (Sigma Aldrich, Vienna, Austria) and washing steps with TBS-T (20 mM Tris-HCl pH 7.6, 150 mM NaCl, 0.1% (*v/v*) Tween 20), the membrane was blocked for 1 h with 5% non-fat dry milk (Carl Roth, Karlsruhe, Germany) diluted in TBS-T. Membranes were incubated with primary antibodies diluted in TBS-T at 4 °C overnight (primary antibodies: anti-IDO-1 (Cell Signaling Technologies, Frankfurt, Germany) 1:500, anti-β-tubulin (Sigma-Aldrich, Vienna, Austria) 1:5000). After washing with TBS-T, peroxidase-conjugated antibodies secondary (Jackson ImmunoResearch Europe Ltd., Cambridgeshire, UK) were added at a dilution of 1:20,000 for 1 h at room temperature (22 °C). After extensive washing with TBS-T, the detection followed using ECL Western blot detection reagent (GE Healthcare, Munich, Germany) on an ImageQuant LAS 4000 (GE Healthcare, Munich, Germany).

### 2.9. Quantitative Real-Rime PCR (qPCR)

Total RNA was isolated by using the RNeasy MiniElute Cleanup Kit (Qiagen, Hilden, Germany) following the manufacturer’s protocol. For the analysis on a Rotor Gene 6000 cycler (Qiagen, Hilden, Germany) 16 ng RNA, specific primers (500 nM each) and the SensiFAST SYBR Lo-ROX One-Step Kit (Bioline, London, UK) were used applying the following conditions: 95 °C 180 s; 40 cycles: 95 °C 5 s, 60 °C 11 s, 72 °C 5 s (acquiring on Sybr Green). To verify primer specificity, a melting curve analysis was performed, and amplicon length was controlled via gel electrophoresis. For each primer pair, the PCR amplicon was verified by sequencing in one experiment. All applications of different cDNA input (*n* = 3, *n* = 4) were performed in duplicates. The ribosomal protein L37a (RPL37A) was used as endogenous control for normalization. Primer sequences were the following: TNF ENSG00000232810 fwd ATGTTGTAGCAAACCCTCAAGC rev AGAGGACCTGGGAGTAGATG; IL1B ENSG00000125538 fwd CCTAAACAGATGAAGTGCTCC rev GAAAGAAGGTGCTCAGGTCAT; IL6 ENSG00000136244 fwd ATTCAATGAGGAGACTTGCCT rev GCTTGTTCCTCACTACTCT; NFKB1 ENSG00000109320 fwd CTCGCCACCCGGCTTCAG rev AGTGCCATCTGTGGTTGAAATA; IDO1 ENSG00000131203 fwd CAGAGGAGCAGACTACAAGAAT rev TAGATTTTCCTGTGGATTTGGCA.

Relative expression ratios (R) of target genes were determined based on the normalized Ct deviation of target gene expression in treated cells versus the control cells according the mathematical model described by M. Pfaffl: ratio = (2^ΔCttarget(control-treated))/(2^ΔCtRPL37A (control-treated)) [37]. The Ct value is defined by the cycle at which the threshold is crossed, and ΔCt is the crossing point difference between sample and control. The relative expression software tool REST 2008 (Technical University of Munich, Germany) was used for statistical analysis [38]. The resulting hypothesis test value P(H1) is an indicator of probability that the difference between sample and control group is significant.

### 2.10. In-Silico Docking Experiments

A computational analysis of possible direct molecular interactions between kaempferol and the active site of IDO-1 was performed by using the crystal structure of human IDO-1 in complex with 4-phenylimidazole as a receptor structure [39]. For docking, the co-crystallized ligand was removed from the active site and protonated chain A of the X-ray structure using MOE’s protonated function [40]. Genetic Optimization for Ligand Docking (GOLD) was used to predict a binding mode for kaempferol in the active site of IDO-1, as GOLD is known to accurately reproduce known protein-ligand geometries for a large range of complexes [41].

Genetic Optimization for Ligand Docking (GOLD) employs a genetic algorithm to explore possible protein–ligand geometries including ligand and partial protein flexibility. Standard settings of GOLD version 5.0.1 were used, if not stated otherwise. The cavity center was set to the position of the co-crystallized ligand 4-phenylimidazole with a cavity radius of 10 Å, hence allowing the full binding pocket to be explored. Ten independent protein-ligand poses were produced to capture possible different binding site orientations. Protein–ligand complex structure visualizations shown in were created with PyMOL (DeLano Scientific LLC, San Francisco, CA, USA, 2008) based on the crystal structure of IDO-1 with 4-phenylimidazole [39].

### 2.11. Statistical Analysis

For statistical analysis, the IBM SPSS software for Windows, version 24 (IBM Corporation, Armonk, USA), was used. For comparison of grouped data, the Kruskal–Wallis test and Mann–Whitney U test were applied. For the cellular antioxidative activity (CAA), assay non-parametric testing was performed (Friedman and Wilcoxon signed-rank tests), as not all data showed normal distribution. Differences were considered to be of significance if p-values were ≤ 0.05. 

The half maximal inhibitory concentration (IC50) was assessed using the CalcuSyn software, version 1.1.1 (Biosoft, Cambridge, UK), according to the concept of Chou and Talalay [42].

## 3. Results

### 3.1. Kaempferol Increases Resistance to Oxidative Stress

The ability of kaempferol and the glycoside kaempferol 3-O-β-D-glucopyranosyl (1→2)-α-L-rhamnopyranoside to counteract intracellular oxidative stress triggered by the peroxyl radical generator AAPH was determined using the keratinocyte cell line HaCaT. The structurally related well-known antioxidant quercetin was applied as positive control.

**Figure 1:**
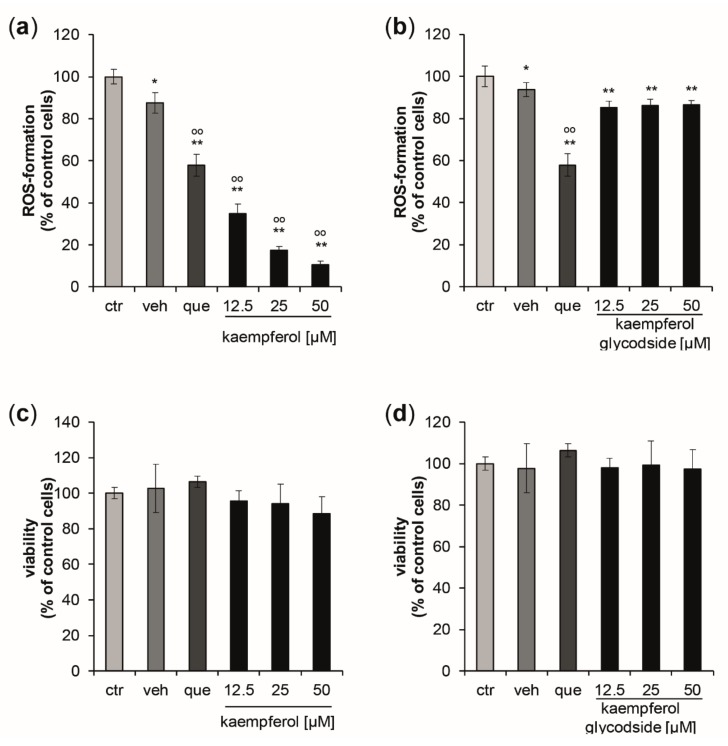
(**a**,**b**) Inhibition of peroxyl-radical-induced reactive oxygen species (ROS)-formation induced by AAPH stimulation in HaCaT keratinocytes pretreated with either quercetin [10 µM] (que) as a positive control, with increasing concentrations of kaempferol, kaempferol glycoside, or with buffer (ctr) or vehicle (veh). ROS formation was calculated as mean percentages of dichlorofluorescein fluorescence, the signal of the cells treated with the radical generator only (ctr = control) was set to 100%. (**c**,**d**) Effects of increasing concentrations of kaempferol and kaempferol glycoside treatment on cell viability measured 24 h after the treatment. Data were normalized to the control cells (ctr), which were treated with buffer only. (Mean values ± S.E.M., *n* = 4, * *p* < 0.05, ** *p* < 0.005 compared to ctr, ° *p* < 0.05, °° *p* < 0.005 compared to veh).

Kaempferol demonstrated significant and dose-dependent antioxidative properties (Figure 1a), leading to a maximum reduction of ROS-formation of 89.4% compared to control cells, with a treatment concentration of 50 µM. The estimated IC_50_ for intracellular ROS inhibition was 7.58 µM, (upper and lower 95% confidence interval (CI_95_) = 6.97 µM and 8.25 µM; correlation coefficient (*R*) = 0.998). Kaempferol glycoside exhibited only scarce ROS scavenging properties, with a maximum reduction of oxidative stress levels by 8.36 ± 2.84% compared to the respective control cells. No dose-dependency was observed (Figure 1b). At the tested concentrations, no significant changes in cell viability were observed for both compounds (Figure 1c,d).

### 3.2. Kaempferol Suppresses Trp Breakdown in Stimulated PBMC and THP1-Blue(-CD14) Cells

**Figure 2:**
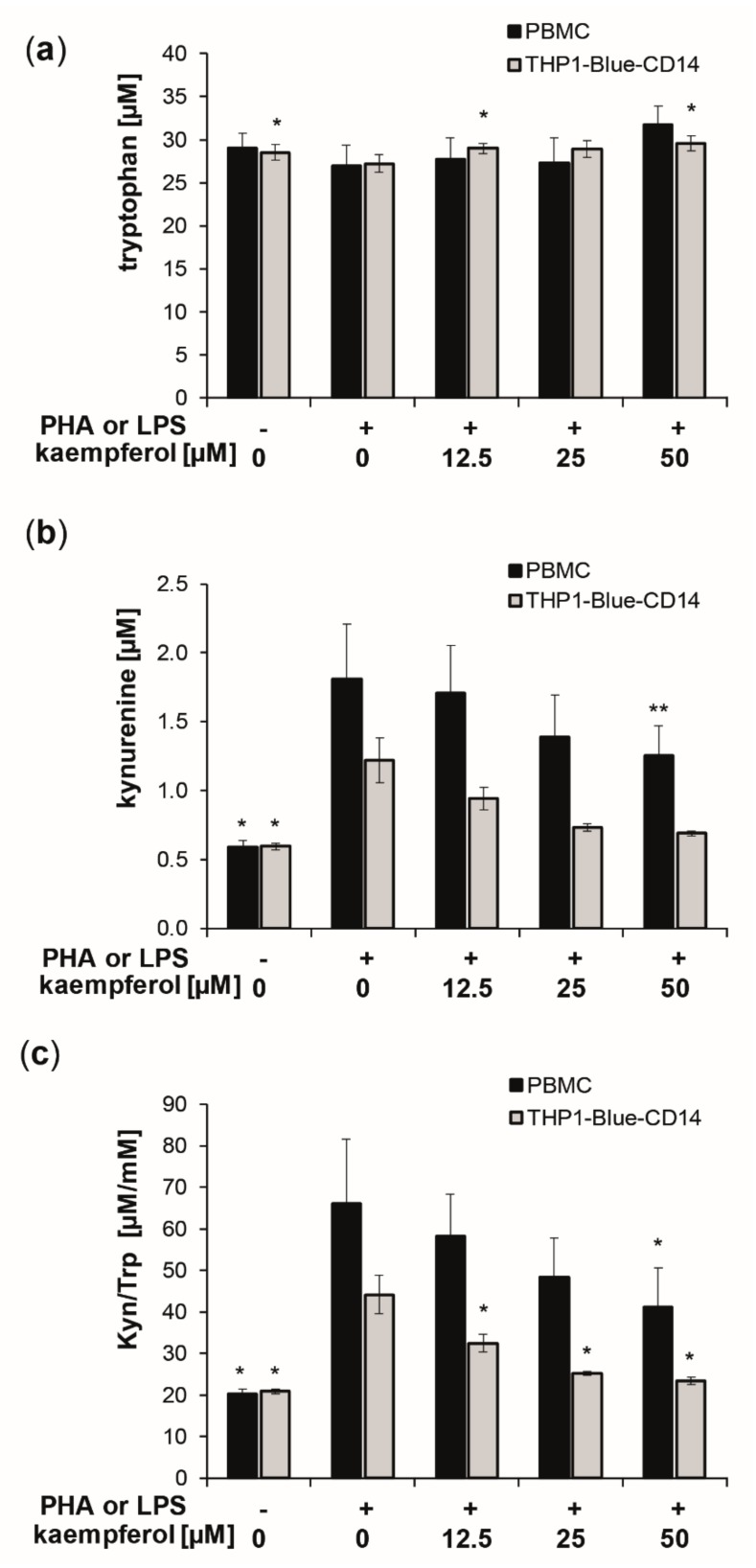
Kaempferol treatment of phytohemagglutinin (PHA) [10 µg/mL]-stimulated human peripheral blood mononuclear cells (PBMC) (black bars) and lipopolysaccharide (LPS) [100 ng/mL]-stimulated THP1-Blue-CD14 (grey bars). Effects on tryptophan (Trp) (**a**) and kynurenine (Kyn) (**b**) concentrations and the Kyn/Trp ratio, a measure of indoleamine 2,3-dioxygenase activity (**c**), was determined in cell culture supernatants after 24 h of incubation. Control cells received neither PHA, LPS, nor kaempferol. (Mean ± S.E.M, *n* = 5 for PBMC and *n* = 4 for THP1-Blue CD14, * *p* < 0.05, ** *p* < 0.005, compared to stimulated control cells).

To evaluate the effect of kaempferol on inflammation-induced Trp breakdown, the concentrations of Trp and its downstream metabolite Kyn, as well as of the oxidative stress marker neopterin, were determined in the supernatants of cells after treatment. The standard culture medium contained a mean Trp concentration of approximately 37 μM. After stimulation of PBMC with PHA and of THP1-Blue-CD14 with LPS, the cells were treated with increasing concentrations of kaempferol [12.5 to 50 µM] for 24 h. Stimulation with PHA and LPS, respectively, increased Trp breakdown, as reflected by increased Kyn concentrations and an elevated Kyn/Trp (Figure 2). 

In the supernatants of PHA stimulated PBMC, Kyn concentrations of 1.8 ± 0.4 µM were measured compared to 0.6 ± 0.0 µM in the untreated PBMC, and 1.2 ± 0.2 µM in stimulated compared to 0.6 ± 0.0 µM in stimulated supernatants of THP1-Blue-CD14 cells (Figure 1b). This resulted in a 3.2-fold elevation in PBMC and a 2.1-fold increase of the Kyn/Trp ratio in THP1-Blue-CD14 cells, reaching values of 66.2 ± 15.4 µM/mM (PBMC) and 44.2 ± 4.6 µM/mM (THP1-Blue-CD14) compared to 20.3 ± 1.1 µM/mM (PBMC) and 20.9 ± 0.5 µM/mM (THP1-Blue-CD14) in unstimulated cells.

Kaempferol treatment did not affect Trp concentrations significantly in PBMCs, though levels were slightly, but not significantly, elevated with the highest treatment concentration of 50 µM. THP1-Blue-CD14 showed a small but significant increase in Trp concentrations at 12.5 µM and 50 µM (Figure 2a). Kyn formation was reduced dose-dependently (Figure 2b). Even more pronounced effects on Trp and Kyn concentrations were observed with longer incubation times (48 h) and also with higher treatment concentrations (see supplementary information: Figure S1). Kyn/Trp decreased significantly and dose-dependently in stimulated PBMC and THP1-Blue-CD14 cells upon kaempferol treatment (Figure 2c). With the maximum applied dose of 50 µM kaempferol, Kyn/Trp was lowered to 41.1 ± 9.5 µM/mM in stimulated PBMC cells and to 23.4 ± 0.9 µM/mM in stimulated THP1-Blue-CD14 cells.

**Figure 3:**
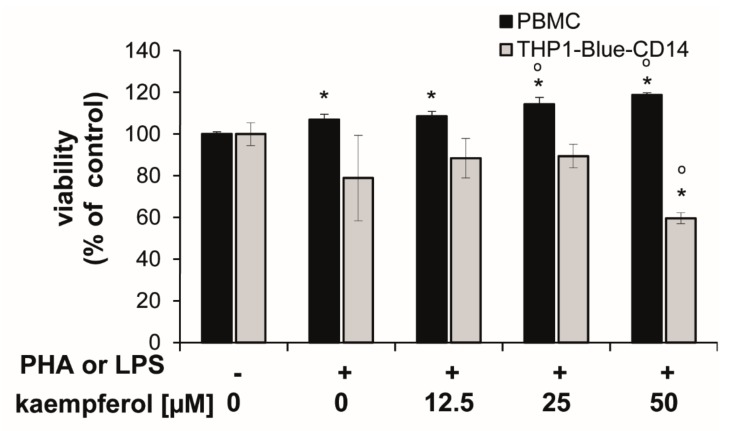
Human peripheral blood mononuclear cells (PBMC) and THP1-Blue CD14 cells stimulated with or without 10 µg/mL phytohemagglutinin (PHA) (for PBMC) or 100 ng/mL lipopolysaccharide (LPS) (THP1-Blue-CD14) prior to incubation with kaempferol for 24 h. Resazurin conversion was determined as a measure for viability. Effect is shown in relation to the activity in the unstimulated controls (set to 100%). Shown are mean values ± S.E.M. of three independent experiments, each performed at minimum in triplicates (* *p* < 0.05 compared to unstimulated control; ° *p* < 0.05 compared to stimulated cells).

Cells showed different sensitivities towards kaempferol treatments at higher concentrations. In stimulated PBMC, the viability was slightly, but significantly increased upon 25 and 50 µM kaempferol addition (approximately 6.8% and 11.0% compared to the PHA-stimulated control cells), respectively. In THP1-Blue-CD14, a viability reduction of 24.4% was observed with a treatment of 50 µM kaempferol in comparison to LPS treated cells (Figure 3).

### 3.3. Reduction of Neopterin Formation by Kaempferol

Upon stimulation with PHA or LPS, neopterin concentrations increased to 3.7 ± 0.4 nmol/L (in PBMC) and 4.3 ± 0.1 nmol/L (in THP1-Blue CD14) after 24 h compared to 2.5 ± 0.3 nmol/L and 2.3 ± 0.1 nmol/L in the respective unstimulated cells.

**Figure 4:**
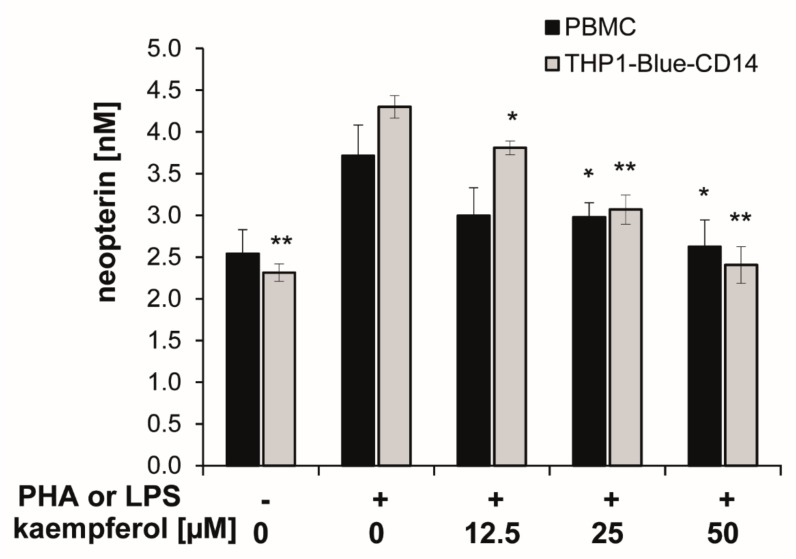
Kaempferol was added to phytohemagglutinin (PHA)-stimulated human peripheral blood mononuclear cells (PBMC) (in black) or lipopolysaccharide (LPS) stimulated THP1-Blue-CD14 cells (in grey). The effects of the different treatments on neopterin formation were determined in supernatants. (mean ± S.E.M, *n* = 5 for PBMC and *n* = 4 for THP1-Blue CD14, * *p* values < 0.05, ** *p* < 0.005 are compared to stimulated control cells).

In both cell models, co-exposure of the stimulated cells to kaempferol inhibited the formation of the oxidative stress marker neopterin in a significant and dose-dependent manner. A treatment concentration of 50 µM kaempferol decreased neopterin levels to 2.6 ± 0.3 nmol/L in stimulated PBMC and to 2.4 ± 0.2 nmol/L in stimulated THP1-Blue CD14 cells, which represents an inhibition of 30% and 44%, respectively (Figure 4). In unstimulated PBMC, however, treatment with increasing concentrations of kaempferol had no major influence on neopterin formation, even if treated with 100 µM of the phytochemical, as shown in the supplementary Figure S1.

### 3.4. In-Silico Docking of Kaempferol to the Active Site of Indoleamine 2,3-Dioxygenase 1 (IDO-1)

**Figure 5:**
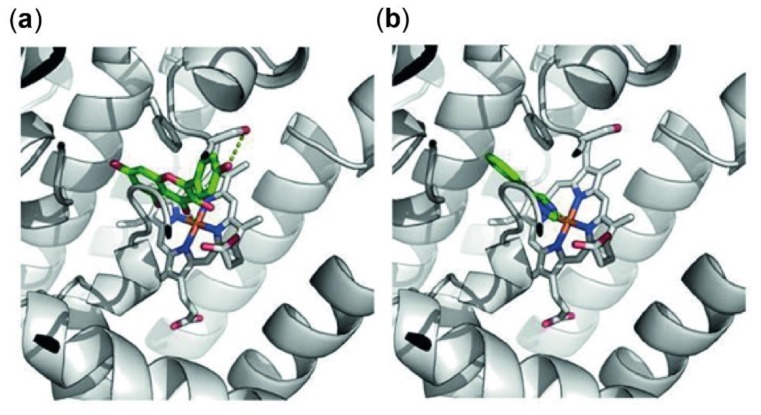
Predicted protein ligand interactions of kaempferol with indoleamine 2,3-dioxygenase 1 (IDO-1) (**a**) and within the crystal structure of 4-phenylimidazole and IDO-1 (**b**). IDO-1 is shown as grey cartoons, the heme group is highlighted with sticks. Ligand molecules are shown in green with polar intermolecular contacts highlighted with yellow dots. Both ligands occupy a similar position in the binding site, both coordinating to the iron of the heme group and stacking to phenylalanine (Phe)-163. Kaempferol shows an additional hydrogen bond to Serine (Ser)-235 not possible in 4-phenylimidazole due to the lack of polar groups.

Downregulation in Trp breakdown can be triggered by direct inhibition of the enzyme IDO-1, that metabolizes the essential amino acid Trp to Kyn, or indirectly by changes in the redox status of the cells or by suppression of the expression of this enzyme on protein or mRNA level. To investigate the mode of action of kaempferol, an in-silico docking study was performed first. Genetic Optimization for Ligand Docking (GOLD) predicted a single favorable binding mode for kaempferol to the active site of IDO-1. All ten predicted protein–ligand complexes were virtually indistinguishable and hence showed consistent protein–ligand interactions. Furthermore, these interactions, as well as the positioning of the ligand in the active site, were highly similar to 4-phenylimidazole as original ligand of the receptor structure of IDO-1. Figure 5 shows details on the active site of IDO-1, showing the similarity between binding modes of kaempferol (Figure 5a) and 4-phenylimidazole (Figure 5b).

The observed binding site orientation of kaempferol shows several favorable protein-ligand interactions: the iron of the active site heme group is tightly coordinating the kaempferol as also observed for 4-phenylimidazole. The aromatic ring system is positioned via aromatic stacking interactions with the benzene ring of Phe-163, as well as additional Van der Waals contacts with Ser-263 and Ala-264. Polar groups of kaempferol are hydrogen-bonded to the hydroxy function of Ser-235 as well as Ser-167. Obviously, the template ligand 4-phenylimidazole lacks these interactions due to the absence of free polar groups. Kaempferol does not show particular interactions with Cys-129, as the compound is too small to protrude these regions of the binding site [43]. Nevertheless, the herein presented model shows that kaempferol occupies a similar position in the binding site compared to that of its known inhibitor 4-phenylimidazole. Consequently, the direct enzymatic inhibition by kaempferol might have to be considered as an important factor when elucidating the mode of action of this natural product.

### 3.5. Effect of Kaempferol on IDO-1 Protein Expression

**Figure 6:**
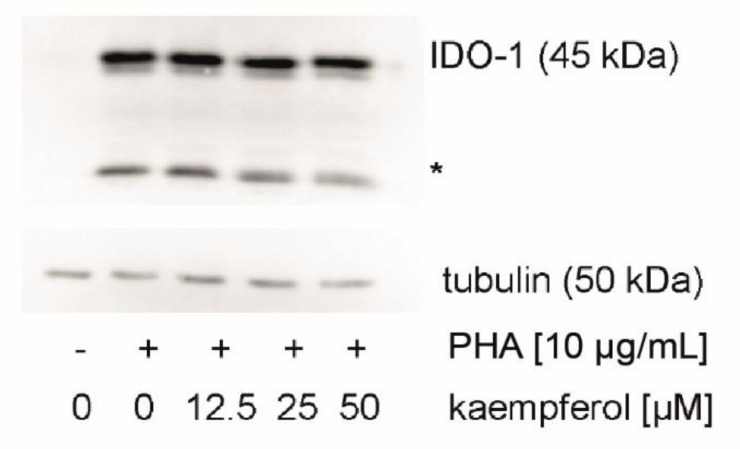
Indoleamine dioxygenase 1 (IDO-1) protein expression was investigated in unstimulated, stimulated, and kaempferol treated peripheral blood mononuclear cells (PBMC). A representative blot is shown, the experiment four times with PBMC obtained from different donors. An additional signal was observed at 25 kDa (indicated with an asterisk). All blots can be found in the supplement (Figures S2 and S3).

To evaluate the effect of kaempferol on the protein levels of the Trp metabolizing enzyme IDO-1, expression levels were analyzed by Western blotting. IDO-1 expression was low in unstimulated cells and increased upon treatment with PHA in PBMC cells (Figure 6). In addition to the expected signal at 45 kDa, a signal of yet unknown origin was observed at 25 kDa, which was reported also by others [44]. No effect of kaempferol treatment on protein levels could be detected with kaempferol concentrations that did not impair the viability of cells. 

### 3.6. Activation of NF-κB/AP-1 by Kaempferol

**Figure 7:**
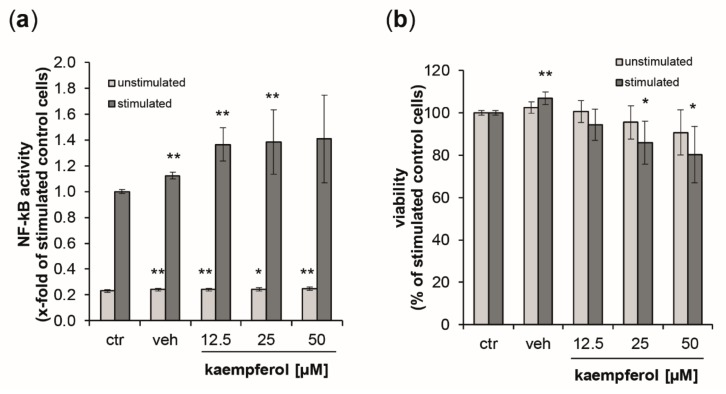
(**a**) Activation of NF-κB/AP-1 (nuclear factor kappa-light-chain-enhancer of activated B cells/activator protein 1) signaling was analyzed in THP1-Blue reporter cells expressing secreted embryonic alkaline phosphatase (SEAP) upon stimulation. Light grey bars represent unstimulated cells, while dark grey bars indicate stimulation with 100 ng/mL of lipopolysaccharide LPS. Cells were left either untreated or were incubated with increasing concentration of kaempferol or the solvent control. SEAP activity was assessed after 24 h. (**b**) Effect of kaempferol on THP1-Blue viability at 24 h post-treatment. Cell viability is shown in comparison to the stimulated buffer control (ctr, set to 100%). Shown are mean values of six independent experiments performed in triplicates (mean ± S.E.M). * *p* values < 0.05 and ** *p* < 0.005 indicate significant changes compared to the respective unstimulated or LPS-stimulated solvent control.

As a measure of NF-κB/AP-1 activity, secreted embryonic alkaline phosphatase (SEAP) levels were estimated in the supernatants of the reporter cell lines THP1-Blue and THP1-Blue-CD14 24 h after the stimulation with LPS (data for THP1blue-CD14 cells can be found in the supplement Figure S4). Stimulation with LPS augmented NF-κB activity by 4.3-fold compared to untreated control cells (Figure 7a). In unstimulated cells, kaempferol, but also the vehicle control induced minor, but significant changes. No dose-dependency could be observed. In the LPS-treated cells, however, SEAP levels were elevated in a dose-dependent manner up to 1.41-fold at 50 µM of kaempferol. For both stimulated and unstimulated cells, the viability decreased in a dose-dependent manner upon kaempferol addition (Figure 7b). LPS-stimulated cells showed a 19.7% decline of viability at the highest tested concentration of kaempferol compared to the stimulated buffer control. This was slightly lower in unstimulated cells, for which a viability reduction of 9.3% compared to the respective control could be observed with 50 µM kaempferol.

### 3.7. Kaempferol Induces Changes in mRNA Levels

LPS-stimulated THP1-blue cells were exposed to 25 and 50 µM kaempferol and the differential expression of IDO-1 and selected NF-κB target genes was analyzed in comparison to the LPS-stimulated, vehicle treated control cells (Table 1). Of note, as mentioned previously, NF-κB activation was observed with both treatment concentrations, but at 50 µM the cell viability was already decreased. 

Treatment with kaempferol did not significantly affect mRNA expression of IDO-1 at both concentrations. Nevertheless, changes in mRNA levels of NF-κB target genes were significant for the nuclear factor kappa B subunit 1 (NF-κB1) which was 2.27-fold and 2.78-fold upregulated with 25 µM and 50 µM kaempferol treatment. The induction of other target genes such as tumor necrosis factor (TNF) and interleukin 1B (IL1B) was significant only at the higher treatment concentration, though at which IL6 expression was reduced. 

## 4. Discussion

The flavonoid kaempferol is highly prevalent in plant-derived aliments and thus is a frequently consumed phytochemical within a balanced diet. Understanding its effect on inflammatory pathways is of great interest, to be able to assess the effectiveness of dietary interventions with kaempferol, which could augment well-being and support health. 

In the present study, it was shown that kaempferol suppressed IDO-1-mediated Trp breakdown as well as neopterin formation efficiently in a dose-dependent manner in mitogen stimulated PBMC and in LPS stimulated human monocytic THP1-Blue-CD14/THP1-Blue cells. Both pathways are activated in parallel upon IFN-γ or LPS stimulation and are highly sensitive towards oxidative triggers. Hence, the antioxidative properties of kaempferol are suggested to play a major role in the immunosuppressive action of this phytochemical, but they are also affecting pro-inflammatory signaling cascades. Data suggest that the inhibitory effect on IDO is mediated by the suppression of the enzyme activity, rather than by affecting mRNA or protein levels. In addition, molecular docking calculations infer that a direct molecular interaction between kaempferol and the enzyme IDO-1 is possible. Changes in the redox status of the cells which interfere with upstream signaling events cannot be excluded, as neopterin formation was also affected. The response of neopterin formation to kaempferol treatment was less prominent, though a direct comparison is not possible (due to unknown differences in response kinetics of metabolite formation). 

A further target of the study was NF-κB signaling, a pathway that is highly influenced by the redox status of the cellular environment, too. ROS are essential modulators in immune responses by determining the balance between apoptotic signaling pathways mediated by TNF and pro-survival pathways activated by NF-κB [45]. It is well-established that LPS-stimulated activation of NF-κB signaling in the THP1-Blue reporter cell line is accompanied by a parallel increase of Kyn and neopterin concentrations, as well as by a decline of Trp [46]. Our results in both THP1-Blue and THP1-Blue-CD14 cell lines confirms these data, the latter being even more sensitive towards LPS stimulation. Rather unexpectedly, kaempferol treatment did not reduce proinflammatory NF-κB activation but even triggered these signaling cascades. It has to be mentioned that both THP1-Blue and THP1-Blue-CD14 cells are more sensitive towards kaempferol treatment than PBMC, as indicated by a reduction of cell viability up to 20% with a treatment concentration of 50 µM. In PHA-stimulated PBMC, 24 h kaempferol treatment resulted in a slight but significant increase in viability compared to PHA treatment alone, however, after 48 h this effect was no longer present (data not shown). Probably, the antioxidant properties of kaempferol provides some advantage for proliferation at an earlier phase of PBMC activation. When proinflammatory signaling becomes more effective and the shift towards an oxidative milieu becomes overwhelming, the protective effect of kaempferol may be consumed.

THP1-Blue/ THP1-Blue-CD14 cells are myelomonocytic tumor cells, while PBMC originate from healthy donors comprising various frequencies of lymphocytes including T cells and monocytes, but also B cells, NK cells, and dendritic cells. Differences in redox homeostasis between the cell models may therefore be expected. Tumor cells are very susceptible to alterations of the redox balance, and earlier studies have shown that kaempferol is very effective to down-regulate the proliferation of various tumor cell lines (see Review by Imran M, et al. [47]).

Interestingly, a superinduction of NF-κB activity in kaempferol plus LPS treated cells compared to LPS treatment could be observed at sublethal concentrations already. Transcription of pro-inflammatory target genes, such as TNF and IL1B, was significantly increased upon 50 µM kaempferol treatment only. At this concentration the expression of IL6 was also reduced. NFKB1 expression, however, was already elevated at the lower concentration of 25 µM. Whether this indicates that at different concentrations distinct gene sets are targeted, e.g., supporting prosurvival and cytoprotective processes, has to be evaluated in the future. Furthermore, also the time of mRNA harvest (24 h after treatment) might have an influence on the activated target genes [48]. Of note, these findings were unexpected and contradict the observations of others that report a suppressive effect on NF-κB signaling (reviewed in Calderón-Montaño et al. [6]). 

Moreover, other enzymes are also known to play important roles in inflammation, such as cyclooxygenase, lipoxygenase, and inducible nitric oxide synthase which are modulated by kaempferol treatment in different cell models [6].

**Figure 8:**
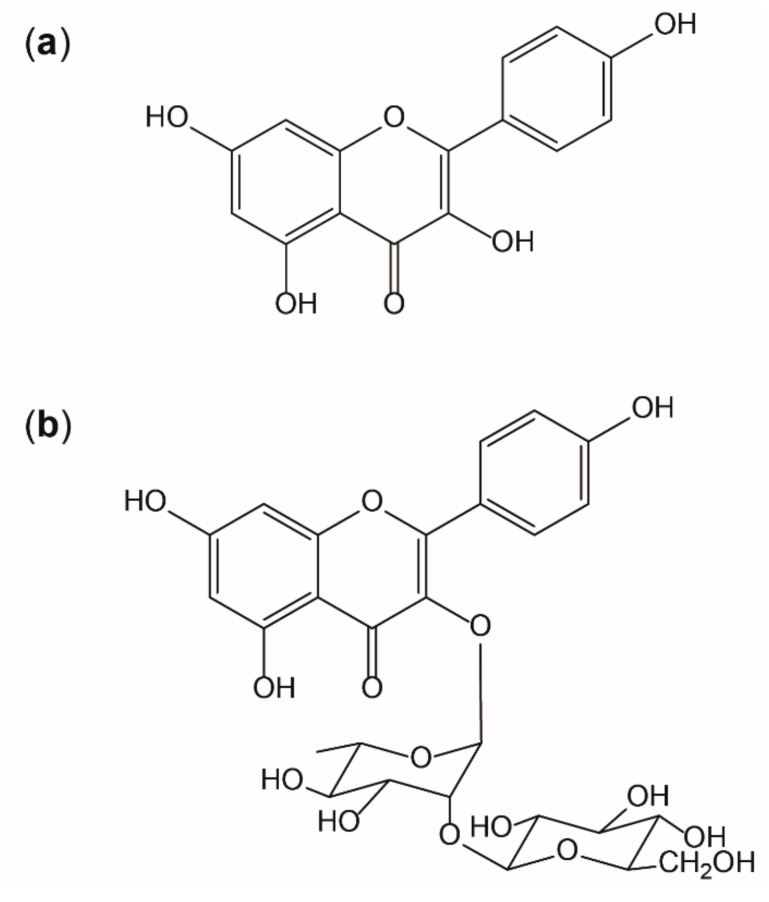
Structure of kaempferol (**a**) and the glycoside kaempferol 3-*O*-β-D-glucopyranosyl (1→2)-α-L-rhamnopyranoside (**b**).

It is known that the interplay of radicals with endogenous and exogenous antioxidants is complex, but essential for the maintenance of physiological functions. However, situations of overwhelming radical formation like those found in inflammation can provoke alterations on molecular and cellular level, if adequate antioxidative countermeasures fail. [9] Thus, antioxidants like kaempferol are thought to be beneficial by supporting endogenous mechanisms [6]. Flavonoids are potent antioxidants in nature as their activity is given by three main aspects: the 3′,4′-dihydroxyl groups on the B-ring, the 2,3-doublebond combined with the 4-keto group on the C ring, and the 3-hydroxyl group also on the C-ring. Kaempferol or 3,5,7-trihydroxy-2-(4-hydroxyphenyl)-4H-1-benzopyran-4-on) (as shown in Figure 8a) (IUPAC) shows all of these structural motives that contribute to antioxidative properties, except of the 3′-hydroxyl group, which, for example, is present in quercetin. This functional group, on the other hand, reduces lipophilicity and therefore quercetin is less likely to pass absorption barriers [49]. In this study, it was shown that the presence of a sugar moiety (like shown in Figure 8b) decreases the bioavailability in vitro.

In humans, kaempferol is known to be metabolized in the intestine [50] and the liver [51], reaching plasma concentrations only in the nanomolar range [52]. Nevertheless, it has still shown biological activities in vivo in several studies [6].

The synergistic or independent action of kaempferol, but also its bioactive metabolites like quercetin, its active transport and facilitated diffusion into tissues, as well as the transportation of this phytochemical in the lymph, might play an essential role for its bioactivity [53]. Moreover, flavonols are known to induce changes in the composition of the gut microbial community, which might be of importance considering that interindividual variations in intestinal microflora can influence disease risks [51,54]. In particular, in the gastrointestinal tract, where kaempferol concentrations are locally higher, the associated lymphoid tissue and immune cell will be exposed to these elevated concentrations. Thus, the relatively low plasma concentrations of kaempferol are probably not a critical limiting factor for its bioactivity, as discussed previously [55,56].

Depending on the desired effects of the treatment, alternative routes of application like dermally or i.v. might also pose interesting options to overcome low oral bioavailability and to reach higher concentrations of kaempferol. The positive effect of a kaempferol-rich diet on human health has been shown in epidemiological studies already [6]. Unfortunately, there are hardly any data on which dose of kaempferol might be safe and tolerable in humans when administered as a single phytochemical. In animal studies, oral kaempferol doses up to 250 mg/kg body weight were administered, and intravenous doses of up to 25 mg/kg body weight) resulting in plasma concentrations of 0.1 to 10 µg/mL [57].

## 5. Conclusions

In summary, results show that this phytochemical contained in everyday food influences redox-sensitive immunomodulatory pathways like IDO-1, GTP-CH-I, and also NF-κB activity, which will all contribute to its bioactivity and underline the pharmacological potential of phytochemicals contained in everyday food. Both the modulation of upstream signaling events and direct interactions with relevant enzymes play a role. However, the immunosuppressive effects may be more relevant in a T cell dominated context, like in the PBMC model, in which the T cell/macrophage interplay is mimicked [31]. Concerted effects of kaempferol on multiple targets contribute to its anti-inflammatory activity and underline the pharmacological potential of this phytochemical. Thus, kaempferol might be an interesting compound for nutritional interventions.

## Figures and Tables

**Table 1:** Relative quantification of IDO1 and NF-κB1 target gene mRNA expression in THP1-Blue cells treated with different concentrations of kaempferol for 24 h, in comparison to LPS plus vehicle treated control cells (expression set as 1).

	25 µM Kaempferol		50 µM Kaempferol	
Target	Relative Expression (95% C.I.)*	P(H1)†	Relative Expression (95% C.I.)*	P(H1)†
IDO1	4.28 (2.06–11.70)	0.072	1.44 (0.63–4.56)	0.557
TNF	5.72 (3.13–12.71)	0.072	6.35 (1.94–17.86)	<0.001
IL1B	4.30 (2.29–10.73)	0.083	4.00 (1.58–11.28)	0.030
IL6	2.68 (1.22–5.62)	0.072	0.31 (0.12–0.76)	0.050
NFKB1	2.27 (1.33–4.05)	<0.001	2.78 (1.49–6.96)	<0.001

* C.I. = confidence interval; † P(H1) = hypothesis test *p* value, significant if ≤ 0.05; *n* = 3.

## Supplementary Materials

The following are available online at https://www.mdpi.com/2076-3921/9/2/180/s1, **Figure S1:** Kaempferol was added to unstimulated (white circles) or PHA-stimulated (black triangles) human PBMC. Its effect on tryptophan (**a**) and kynurenine (**b**) concentrations, the kyn/trp ratio (**c**) and neopterin levels (**d**) was determined in the cell supernatants after 48 h of incubation. Kynurenine and neopterin concentrations are expressed as % of baseline (control cells treated with or without PHA). Tryptophan concentrations are expressed as % of medium control. (Mean ± S.E.M, *N* = 3, * *p* < 0.05, compared to baseline). **Figure S2:** Western blots (full-size) of unstimulated, stimulated and kaempferol treated PBMCs incubated with antibodies against indoleamine dioxygenase 1 (IDO-1) protein (upper) and tubulin (lower blot). A representative of PBMC from two different donors is shown. An additional signal of yet unkown origin was observed at 25 kDa (indicated with an asterisk). **Figure S3:** PBMC were isolated in four independent experiments from different healthy donors to analyse indoleamine dioxygenase 1 (IDO-1) expression after treatmen t of the cells with PHA and kaempferol. An additional signal of yet unknown origin was observed at 25 kDa (indicated with an asterisk). **Figure S4:** (**a**) NF-κB/AP-1 activation was estimated in unstimulated (light grey) and stimulated (100 ng/mL LPS, dark grey) THP1-Blue-CD14 reporter cells. Cells were left either untreated, or were incubated with increasing concentrations of kaempferol, or the solvent control (veh) for 24 h. (**b**) Effect of kaempferol on THP1-Blue-CD14 viability at 24 h post-treatment. Cell viability is shown in comparison to the stimulated buffer control (ctr, set to 100%). Shown are mean values of three independent experiments performed in triplicates (mean ± S.E.M). **p*-values < 0.05 indicate significant changes compared to the respective unstimulated or LPS-stimulated solvent control.
