# Supplementary material for: Pharmacological Targets of Kaempferol Within Inflammatory Pathways—A Hint Towards the Central Role of Tryptophan Metabolism
Source: Antioxidants (Basel). 2020 Feb 21;9(2):180. doi: 10.3390/antiox9020180 (PMC7070836; doi:10.3390/antiox9020180)
Supplement: Supplementary file 1 [file antioxidants-09-00180-s001.pdf]

Supplementary material

Pharmacological targets of kaempferol within inflammatory pathways – a hint towards the central role of tryptophan metabolism

Stefanie Hofer[1,2], Simon Geisler[3], Rebecca Lisandrelli[1], Hieu Nguyen Ngoc[2], Markus Ganzera[2], Harald Schennach[4], Dietmar Fuchs[3], Julian E. Fuchs[5], Johanna M. Gostner[1] and Katharina Kurz[6*]

[1] Institute of Medical Biochemistry, Biocenter, Medical University of Innsbruck, Innrain 80, 6020 Innsbruck, Austria;

[2] Institute of Pharmacy/Pharmacognosy, University of Innsbruck, Innrain 80 - 82/IV, 6020 Innsbruck, Austria;

[3] Institute of Biological Chemistry, Biocenter, Medical University of Innsbruck, Innrain 80, 6020 Innsbruck, Austria;

[4] Central Institute of Blood Transfusion and Immunology, University Hospital, Anichstrasse 35, 6020 Innsbruck, Austria;

[5] Department of Medicinal Chemistry, Boehringer Ingelheim RCV GmbH & Co KG, Dr. Boehringer-Gasse 5-11, 1120 Vienna, Austria;

[6] Department of Internal Medicine II, Infectious Diseases, Pneumology, Rheumatology, Medical University of Innsbruck, Anichstrasse 35, 6020 Innsbruck, Austria

* Correspondence: katharina.kurz@i-med.ac.at; Tel.: +43-512-504-23260

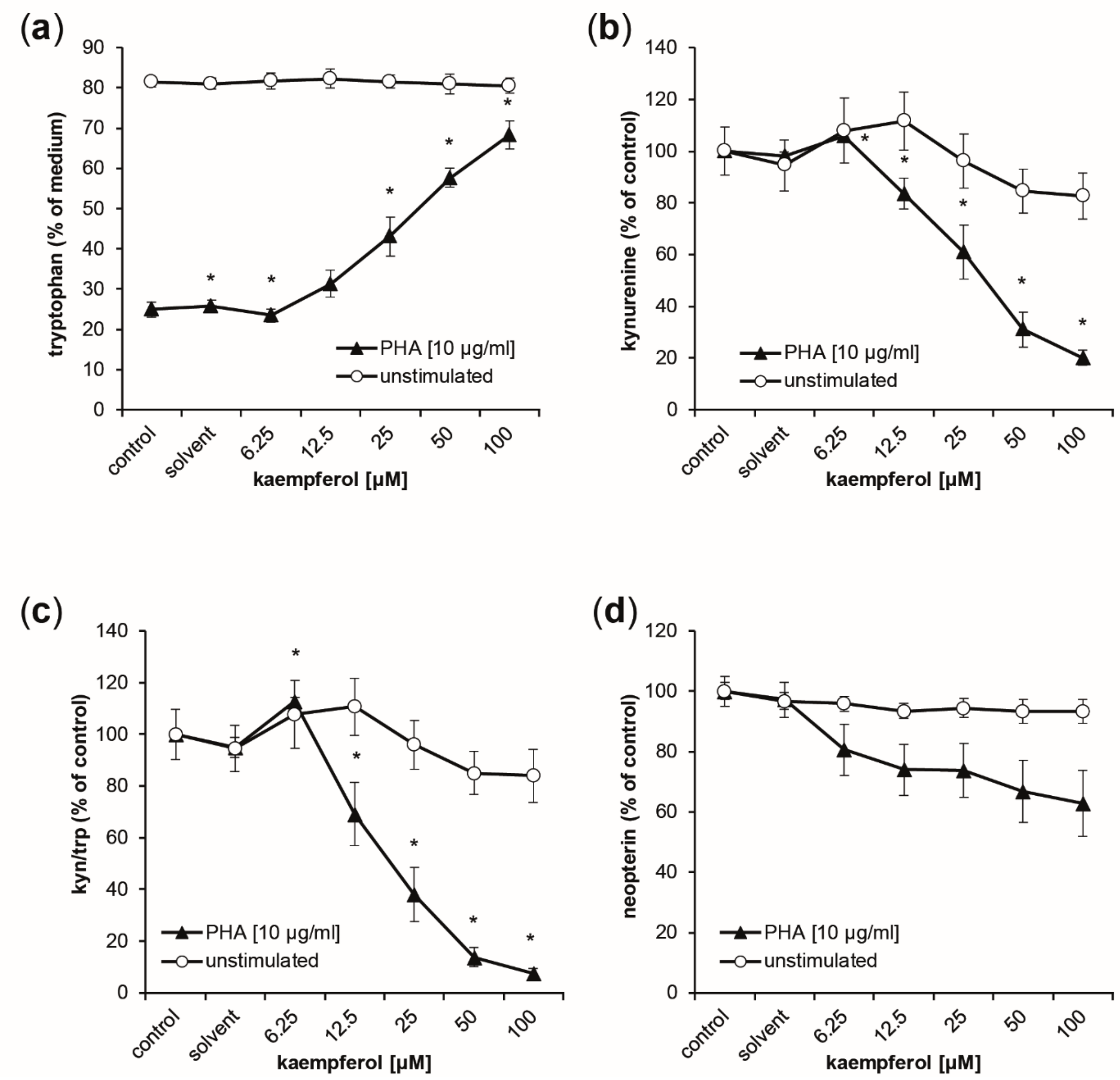

Figure S1: Kaempferol was added to unstimulated (white circles) or PHA-stimulated (black triangles) human PBMC. Its effect on tryptophan (**a**) and kynurenine (**b**) concentrations, the kyn/trp ratio (**c**) and neopterin levels (**d**) was determined in the cell supernatants after 48 h of incubation. Kynurenine and neopterin concentrations are expressed as % of baseline (control cells treated with or without PHA). Tryptophan concentrations are expressed as % of medium control. (Mean ± S.E.M, $N = 3$, $^*p < 0.05$, compared to baseline).

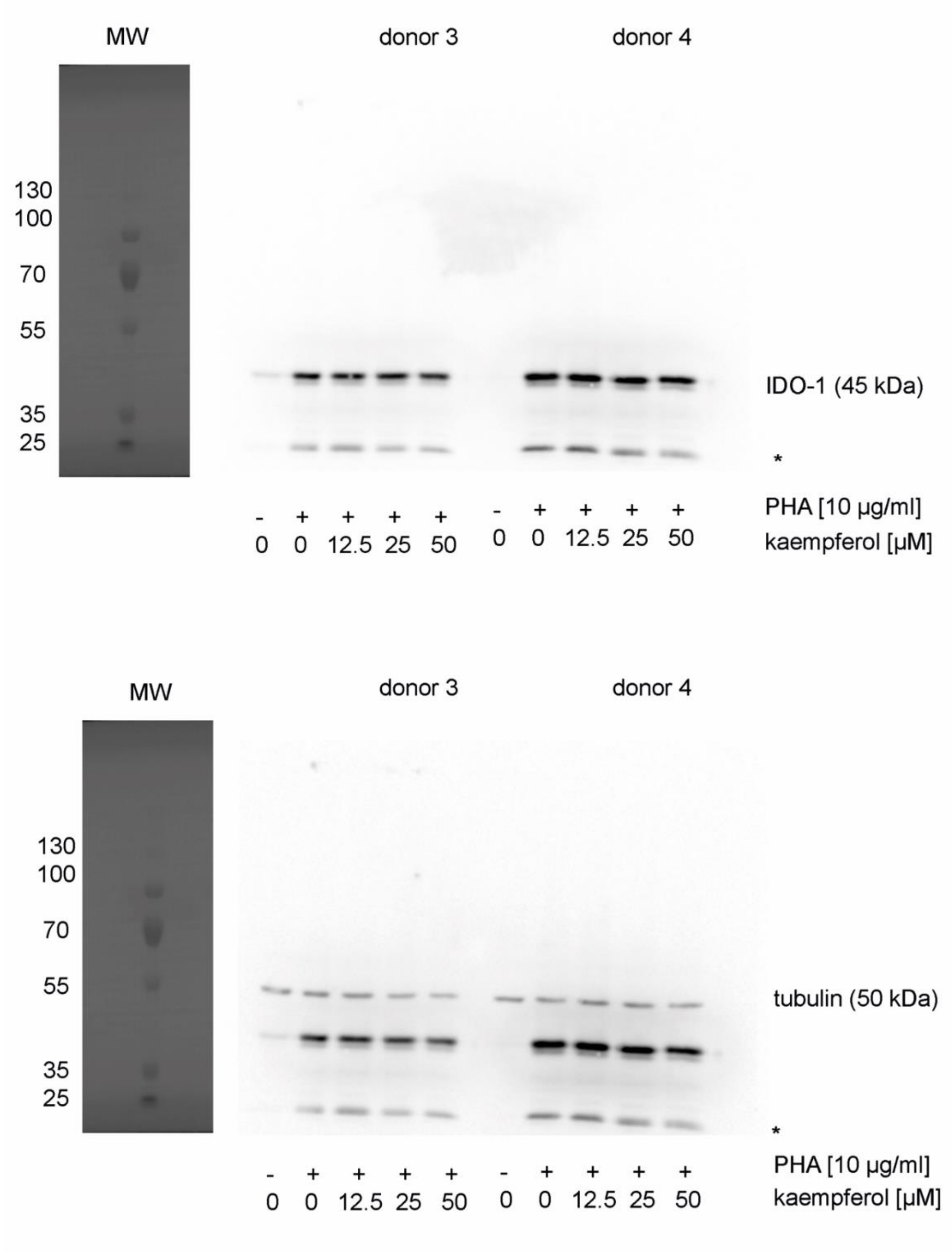

Figure S2: Western blots (full-size) of unstimulated, stimulated and kaempferol treated PBMCs incubated with antibodies against indoleamine dioxygenase 1 (IDO-1) protein (upper) and tubulin (lower blot). A representative of PBMC from two different donors is shown. An additional signal of yet unkown origin was observed at 25 kDa (indicated with an asterisk).

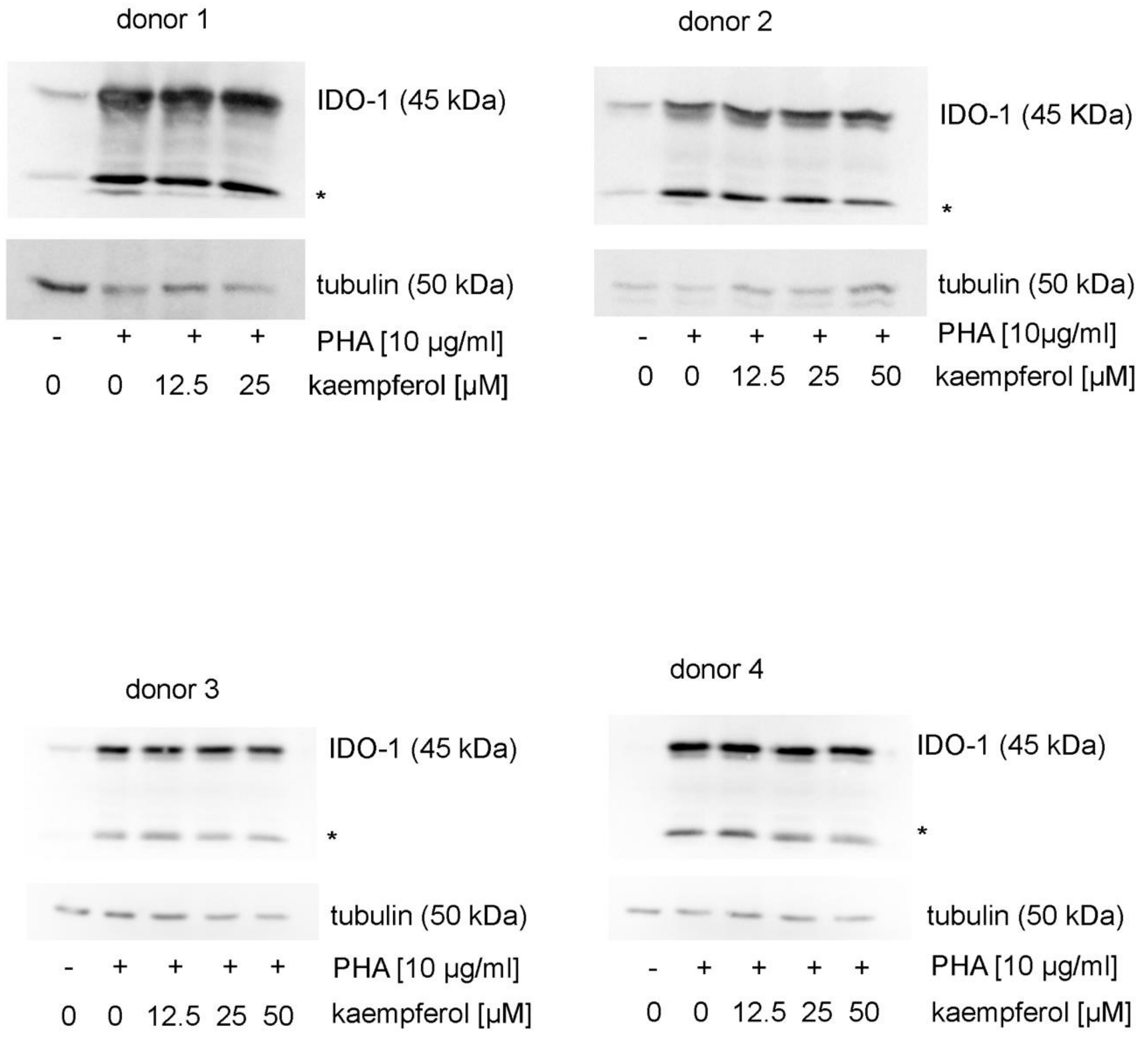

Figure S3: PBMC were isolated in four independent experiments from different healthy donors to analyse indoleamine dioxygenase 1 (IDO-1) expression after treatmen t of the cells with PHA and kaempferol. An additional signal of yet unknown origin was observed at 25 kDa (indicated with an asterisk).

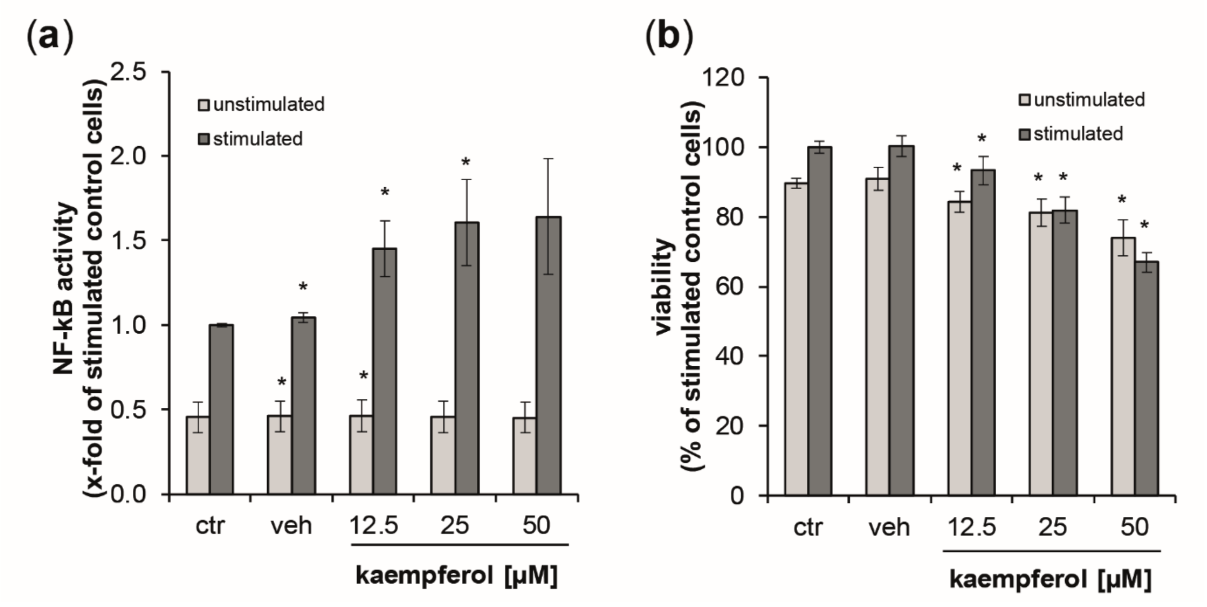

Figure S4: (**a**) NF-κB/AP-1 activation was estimated in unstimulated (light grey) and stimulated (100 ng/mL LPS, dark grey) THP1-Blue-CD14 reporter cells. Cells were left either untreated, or were incubated with increasing concentrations of kaempferol, or the solvent control (veh) for 24 h. (**b**) Effect of kaempferol on THP1-Blue-CD14 viability at 24 h post-treatment. Cell viability is shown in comparison to the stimulated buffer control (ctr, set to 100%). Shown are mean values of three independent experiments performed in triplicates (mean ± S.E.M). *p-values < 0.05 indicate significant changes compared to the respective unstimulated or LPS-stimulated solvent control.
